# Cortical Dynamics of Acoustic and Phonological Processing in Speech Perception

**DOI:** 10.1371/journal.pone.0020963

**Published:** 2011-06-13

**Authors:** Linjun Zhang, Jie Xi, Guoqing Xu, Hua Shu, Xiaoyi Wang, Ping Li

**Affiliations:** 1 State Key Laboratory of Cognitive Neuroscience and Learning, Beijing Normal University, Beijing, China; 2 College of Chinese Studies, Beijing Language and Culture University, Beijing, China; 3 Institute of Psychology, Chinese Academy of Sciences, Beijing, China; 4 Department of Psychology, Dalian Medical University, Dalian, China; 5 Department of Radiology, Xuanwu Hospital, Capital Medical University, Beijing, China; 6 Department of Psychology and Center for Language Science, Pennsylvania State University, University Park, Pennsylvania, United States of America; University of Regensburg, Germany

## Abstract

In speech perception, a functional hierarchy has been proposed by recent functional neuroimaging studies: Core auditory areas on the dorsal plane of superior temporal gyrus (STG) are sensitive to basic acoustic characteristics, whereas downstream regions, specifically the left superior temporal sulcus (STS) and middle temporal gyrus (MTG) ventral to Heschl's gyrus (HG) are responsive to abstract phonological features. What is unclear so far is the relationship between the dorsal and ventral processes, especially with regard to whether low-level acoustic processing is modulated by high-level phonological processing. To address the issue, we assessed sensitivity of core auditory and downstream regions to acoustic and phonological variations by using within- and across-category lexical tonal continua with equal physical intervals. We found that relative to within-category variation, across-category variation elicited stronger activation in the left middle MTG (mMTG), apparently reflecting the abstract phonological representations. At the same time, activation in the core auditory region decreased, resulting from the top-down influences of phonological processing. These results support a hierarchical organization of the ventral acoustic-phonological processing stream, which originates in the right HG/STG and projects to the left mMTG. Furthermore, our study provides direct evidence that low-level acoustic analysis is modulated by high-level phonological representations, revealing the cortical dynamics of acoustic and phonological processing in speech perception. Our findings confirm the existence of reciprocal progression projections in the auditory pathways and the roles of both feed-forward and feedback mechanisms in speech perception.

## Introduction

In speech perception, the brain system is exposed to continuously changing streams of sound, which are complex in spectral and temporal features. Human listeners generally perform this perception task automatically and effortlessly despite the complexities in encoding acoustic characteristics and extracting phonological properties of speech. While the cognitive mechanisms of speech perception are well understood in psycholinguistics, the neural mechanisms underlying how the brain converts speech signals to phonological and semantic representations have been the subject of intensive investigation in recent neuroimaging studies.

There has been converging evidence recently that speech is processed along the auditory pathways in a hierarchical manner. That is, the dorsal STG areas perform initial acoustic analysis, whereas the ventral STS/MTG regions are responsible for phonological processing [1]–[9]. What is unclear from these studies is the relationship between the two processes, especially whether the “top-down” phonological representations influence the “bottom-up” acoustic analysis. Studies in visual cortical processing have revealed that neuronal responses at early stages, including those in primary visual cortex (V1), are subject to task-specific top-down influences [10]–[12]. The importance of top-down influences in sound processing has also been highlighted in recent studies. The majority of the research has so far focused on the influences of attention and task, and found that attention to particular features and active listening tasks modulate activation in the lower level auditory areas [13]–[15]. Therefore, it is generally difficult to infer from these results whether the encoding of acoustic features is modulated by the processing of abstract phonological information. Despite the lack of empirical evidence, the literature contains two opposing views regarding the top-down versus bottom-up interactions: (1) a feed-forward flow of information in speech sound abstraction, according to which unidirectional progression of processing runs from the core auditory areas to the lateral STG and then to the more lateral and anterior regions [7], [16]; and (2) reciprocal projections are involved in speech perception, which allows for feedback connections and significant top-down influences on lower-level processes [9].

To understand the nature of the interaction between acoustic and phonological processing in speech perception, in this study we used functional magnetic resonance imaging (fMRI) to assess sensitivity of core auditory and downstream brain regions to acoustic and phonological variations by using within- and across-category lexical tonal continua with equal physical intervals. On the assumptions of the Hickok and Poeppel model [9], we expected that (1) there would be greater activation in the downstream regions such as STS/MTG elicited by across-category stimuli than within-category stimuli, reflecting the processing of phonological information, and (2) there would be weaker activation in the dorsal auditory areas such as HG/STG elicited by across-category stimuli than within-category stimuli, reflecting the top-down modulation of phonological processing on the bottom-up acoustic analysis. Evidence to the contrary would provide support to the feed-forward models [7], [16], that is, similar activation would be observed in the dorsal STG areas for both within- and across-category stimuli, suggesting simple bottom-up sequences of responses given that both sounds entail similar acoustic analysis.

## Materials and Methods

### Participants

We tested 17 neurologically healthy volunteers (11 females; mean age 22, range 19–25) with normal hearing and minimal musical experience (less than 1 year of total musical training and no musical training within the past 5 years). All participants were native speakers of Chinese and were right-handed.

### Ethics Statement

Written informed consent was obtained from all participants after they were given a complete description of the study. The study was approved by the ethical research committee at Beijing Normal University's Imaging Center for Brain Research.

### Materials

**Figure 1:**
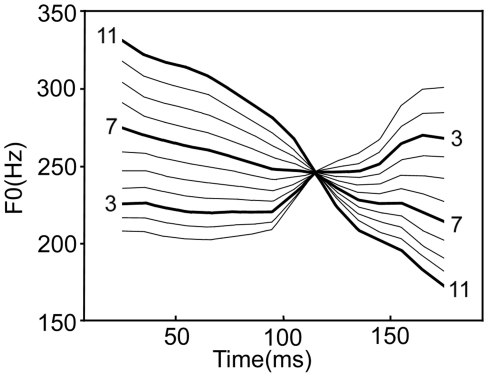
Tone contours of the continuum from /ba2/ to /ba4/. Continua 3, 7 and 11 are marked with thick lines.

The stimuli were previously used in an event-related potential (ERP) study [17]. They were chosen from Chinese lexical tonal continuum from the high-rising tone (Tone2) to the falling tone (Tone4) (Fig. 1), which form an across-category stimulus pair (3 and 7) and a within-category stimulus pair (7 and 11). Because the physical intervals between the across-category pair and within-category pair were equated [17], the key difference between the stimulus pairs is that the former involves a change to a different phonological category, while the latter involves only an acoustic change. As in the ERP study, in our fMRI study we used stimulus 7 as the standard stimulus, and stimuli 3 and 11 as the across-category and within-category deviants, respectively.

### Experimental Design

**Figure 2:**
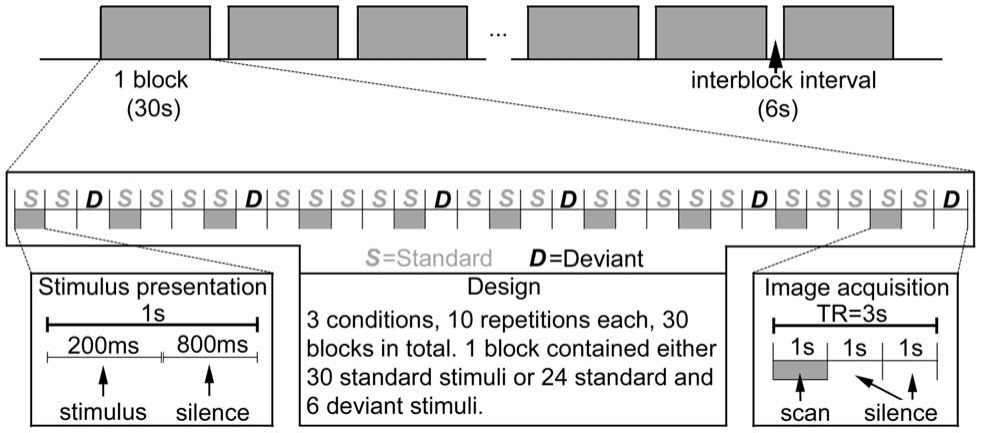
Schematic illustration of the experimental paradigm.

The experimental design (Fig. 2) was adapted from Tervaniemi et al. 's study [18]. The sounds were presented in blocked design with 1 s stimulus-onset asynchrony (SOA). Three conditions of stimuli were presented in separate blocks and each block consisted of either 30 standard stimuli, a mixed sequence of standard stimuli and across-category deviants, or standard stimuli and within-category deviants. In a mixed sequence, there were six deviants presented in a pseudorandom order among the standard stimuli. Because of demands set by the clustered scanning paradigm, deviant stimuli were never presented in the first, fourth, seventh, etc., position. The experiment consisted of 2 scanning runs, 15 blocks each (5 blocks for each condition), that is, a total of 10 repetitions for each condition of stimuli. Each run was preceded by a 9 s silent baseline to familiarize the participants with the situation.

During the scanning, participants viewed pictures through a projection screen reflected on a mirror inside the scanner. The sound stimuli were presented binaurally via SereneSound (Resonance Technology Inc., Northridge, CA, USA) 30 dB external noise attenuating MRI-compatible headphones. Sound levels were adjusted to comfortable levels (70–80 dB) for each subject before the experiment began. All participants reported that they could hear the stimuli and discriminate them from the background scanner noise. Because we were interested in pre-attentive auditory perception effects, participants were told to ignore the presented sounds. The participants were instructed to finish a visual task. Black-and-white drawings of landscape or the same drawings in color were presented, and participants needed to press the left or the right response button with their index finger every time a drawing appeared in color (counterbalanced for left versus right across subjects). The pictures were presented in a random order, with display duration and inter-picture intervals of 1.5 s, 2 s or 2.5 s. The response had to be performed during a 6 s silent interval, which separated the blocks.

### Image Acquisition

Data were acquired on a Siemens Magnetom Trio 3-T scanner at the Beijing Normal University's Imaging Center for Brain Research. Foam pads were used for stabilizing the participant's head within the head coil to restrict movement. During functional scanning, a T2*-weighted gradient-echo echo-planar imaging (EPI) sequence was used to acquire functional images with 16 axial slices (TR/TE  = 3000/30 ms, flip angle  = 90°, slices thickness  = 3 mm, 0.45 mm gap between slices, matrix  = 64×64, FOV  = 200 mm ×200 mm) parallel to the STS covering STG, STS, MTG, insular lobe, thalamus and part of frontal/parietal cortex. The TR was optimized to allow the BOLD signal caused by gradient switching to diminish and to minimize the length of the scanning sessions. Slice acquisition was clustered in the first second of the TR, leaving two seconds without gradient noise and deviant stimuli occurred only during the silent period [18]. A total of 181 volumes were acquired per run, with the first three images being discarded to allow the spins to reach relaxation equilibrium. High-resolution anatomical images (256 slices) of the entire brain were obtained by a 3-D gradient-echo sequence (SPGR) after the functional images were acquired, with the following parameters: matrix  = 256×256, TR/TE  = 2530/3.6 ms, flip angle  = 7°.

### Data Analysis

The imaging data were preprocessed and statistically analyzed using the AFNI software (http://afni.nimh.nih.gov/afni) [19]. The first three scans were excluded from data processing to minimize the transit effects of hemodynamic responses. Functional images were corrected for head motion by aligning all volumes to the fourth volume using a six-parameter rigid-body transformation. The corrected images were spatially smoothed by a 6-mm FWHM Gaussian kernel. Then, the changes of signals on time series about each voxel were detrended and normalized to one hundred. At the individual level, the preprocessed images were submitted to individual GLM-based analyses to estimate the individual statistical t-maps for each participant. There were nine regressors in the General Linear Model in all, with three experimental conditions and six head-motion parameters. The functional t-maps of each participant were registered to his/her high-resolution anatomical image (SPGR) and normalized according to the standard stereotactic space [20]. Thereafter, a three-dimensional data set, rescaled to a voxel size of 2×2×2 mm^3^, was created. At the group level, in a voxel-wise random effects analysis, percent signal changes on the functional t-maps for each condition and each participant were entered into a two-way, mixed-factor ANOVA with condition as fixed factor and participant as random factor. A group statistical map was created, using a structural mask of bilateral temporal lobe with three contrasts: across-category deviant vs. standard, within-category deviant vs. standard, and across-category deviant vs. within-category deviant. The contrast maps were corrected for multiple comparisons at *p*<0.05 (41 contiguous voxels at a voxel-level threshold of *p*<0.005, *t*>2.248) by AlphaSim with an anatomical mask including bilateral superior, middle, inferior temporal gyrus and HG. Regions-of-interest (ROI) analysis was then conducted to examine the nature of the neural responses in areas identified in the within-category deviant vs. across-category deviant contrast. The ROIs were defined functionally as spheres with a 6-mm radius on the basis of activation clusters from the group analysis. We selected the peak activation coordinates from the cluster of the contrast analysis as the center of each ROI. Once selected, the ROIs were employed as masks to extract the mean percent signal change (averaged over the ROI) in the blood oxygen level dependent (BOLD) response.

### Behavioral Posttest

Discrimination experiment on 13 of the fMRI participants was carried out 6 months after scanning (4 participants were not available) in order to ensure that familiarity with the stimuli did not artificially influence task performance [3]. The purpose of the behavioral posttest was to examine whether the mismatch neural responses are congruent with an overt meta-linguistic task. The AX discrimination task required participants to listen over headphones and judge whether the identical (3 vs. 3, 7 vs. 7 and 11 vs. 11), within-category (7 vs. 11), and across-category (3 vs. 7) pairs were the same or different. For the within- and across-category pairs, both directions of presentation order were tested. Each pair was presented 10 times and the stimulus presentation was randomized.

## Results

### Behavioral Posttest

**Figure 3:**
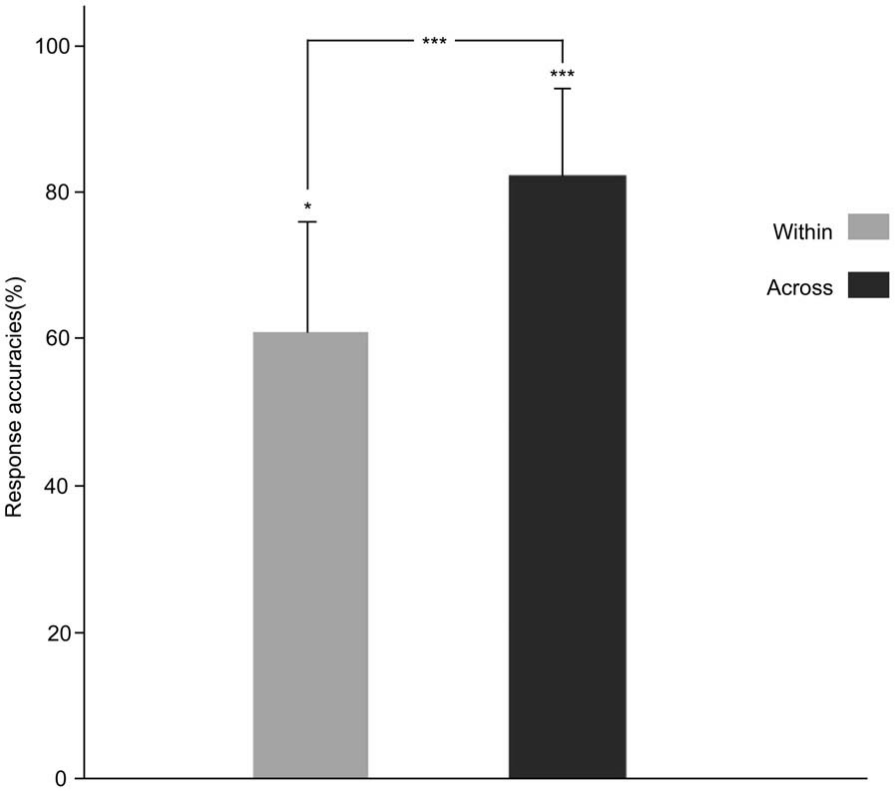
Overt discrimination of stimuli in the fMRI experiment obtained from 13 participants 6 months after scanning. * *p*<0.05, *** *p*<0.001.

Participants' discrimination performance is plotted in Fig. 3, indicating above-chance level discrimination for both within- and across-category pairs (for the within-category pair, *t*(12)  = 2.383, *p* = 0.035; for the across-category pair, *t*(12)  = 9.634, *p* = 0.000). Participants were clearly sensitive to both types of contrasts, although the across-category pairs elicited higher rates of “different” responses compared with the within-category pairs (*t*(12)  = 5.647, *p* = 0.000), reflecting the categorical perception effects.

### Functional Magnetic Resonance Imaging

Table 1 presents a summary of all activation clusters in the planned comparisons: within-category deviant vs. standard, across-category deviant vs. standard, and across-category deviant vs. within-category deviant. Below we focus on the contrasts between across-category deviant vs. within-category deviant conditions.

**Table 1:** Areas of significant activation.

AnatomicalRegion	Brodmann	Peak Voxel Coordinates			Voxels	*t*
	area	*x*	*y*	*z*		
**Within-category > ** **Standard**						
Right STG	22/42	53	−21	−6	648	5.79
Left STG	22	−59	−41	20	113	5.34
Left STG	22	−47	3	2	42	6.17
**Within-category < ** **Standard**						
——						
**Across-category > ** **Standard**						
Right STG	22	59	−47	20	53	4.35
**Across-category < ** **Standard**						
Left STG/HG	41/42	−53	−17	8	66	4.96
**Across- > ** **Within-category**						
Left MTG	21	−53	−25	−10	42	4.69
**Within- > ** **Across -category**						
Right STG/HG	22/41	49	−9	6	363	4.76
Left STG/HG	42/41	−47	−25	14	223	4.66

Note: Areas identified in the group analysis for all the planned comparisons, thresholded at a voxel-wise *P*<0.005 (*t*>2.248). Cluster level activated volume ≥328 mm^3^ (*P*<0.05, corrected). Coordinates are in Talairach and Tournoux (1988) space.

**Figure 4:**
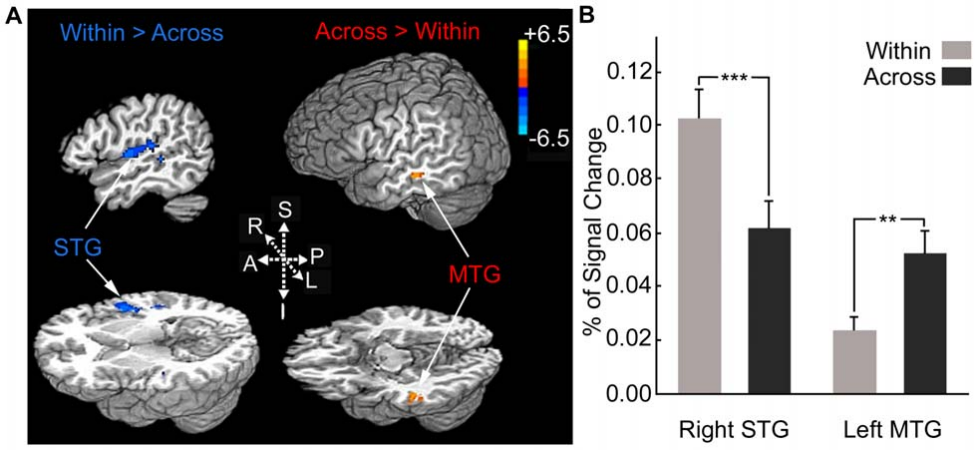
Activation in within-category deviant vs. across-category deviant contrast. (A) Significant foci of activity for within-category deviant > across-category deviant (blue) and across-category deviant > within-category deviant (orange). (B) Mean percent BOLD signal change extracted from ROIs based on functionally defined activation clusters in the left mMTG (x = −53, y = −25, z = −10) and right lateral HG (x = 49, y = −9, z = 6). ** *p*<0.01, *** *p*<0.001.

When contrasting within-category deviant condition to across-category deviant condition, significant activation for the within-category condition was in the STG/HG areas bilaterally. Greater activation for the across-category condition was only observed in the left MTG (Fig. 4*A* and Table 1). To further explore the relationship between the identified areas, we also conducted ROI analysis within the left mMTG and right lateral HG, which were defined functionally on the basis of activation clusters from the group analysis (x = −53, y = −25, z = −10, left mMTG and x = 49, y = −9, z = 6, right lateral HG). The two areas were selected for ROI analysis because of their well-known roles in high-level phonological representations and low-level acoustic, especially pitch analysis, respectively. Paired-samples t-tests were used to compare the mean percent signal changes in the two conditions. In the left mMTG, the across-category deviant condition yielded significantly greater percent signal changes than the within-category deviant condition (*t*(16)  = 3.81, *p* = 0.002), whereas the reverse pattern was observed in the right lateral HG, with greater percent signal changes for the within-category deviant condition than the across-category deviant condition (*t*(16)  = 4.5, *p* = 0.000) (Fig. 4*B*). The two conditions were carefully controlled for effects of acoustic difference, so that the stronger activation in the left mMTG could be interpreted as higher sensitivity to phonological features and the less activation in the right lateral HG as indicating that more accurate acoustic analysis is not necessary when phonological information is present.

## Discussion

In speech perception, the auditory cortex needs to decode complex acoustic patterns and it has been widely accepted that the processes are performed in a hierarchical manner [7]–[9]. The present data are consistent with the proposed functional hierarchy. Acoustic variation (within-category deviant condition > standard condition) elicited more activation in the dorsal and posterolateral superior temporal areas, with the strongest activation in the right middle STG. This pattern matches well with those from previous imaging studies that showed that the right middle STG or nearby regions play an important role in the processing of speech prosody [21]–[24]. In contrast, brain areas specifically activated by phonological variation (across-category deviant condition > within-category deviant condition) were located in the left mMTG. The middle STS and MTG regions, especially in the left hemisphere have been implicated to play specific roles in phonemic perception [2], [6]. These areas lie at a point along the ventral auditory pathway, where abstract phonological representations have already been segregated from detailed acoustic patterns. The anterior portion of the STS and more ventral portion of the temporal cortex, such as the inferior temporal gyri, are associated with sentence-level speech comprehension, including phonetic, semantic and syntactic analysis [25], [26]. Considering the previous findings and the present results together, we suggest that the mSTS/MTG represents an “intermediate” stage of processing, linking areas in the dorsal STG for acoustic processing to areas in the left anterior STS and inferior temporal gyri for higher-level linguistic processes.

Although recent imaging studies have increasingly shown that a dissociation exists between acoustic and phonological processing at different hierarchical levels of the auditory cortex (dorsal STG versus mSTS/MTG) and in different hemispheres (right versus left), the manner in which these regions interact during speech perception is not well understood. By comparing activation elicited by acoustically different types of intelligible speech relative to unintelligible controls of similar acoustic complexity, previous imaging studies isolated brain areas that respond specifically to intelligibility [27]–[29]. In these studies, however, brain regions which are sensitive to acoustic features only were not found. Using a multivariate pattern analysis, a recent fMRI study showed that the auditory core regions and the posterior STS are sensitive to acoustic and phonological features respectively [5]. The interaction between these brain processes, however, was not explored because the speech and nonspeech control stimuli were not acoustically comparable.

In the current study, we used lexical tonal continua with equal physical intervals, which allows us to test directly whether there is interaction between acoustic and phonological processing. Our data show that compared with acoustic variations, phonological variations elicited less activation in the core auditory region, exactly the right lateral HG, which has been suggested to function as a “pitch center” [30], [31] that is specifically responsive to pitch sequences in speech or non-speech context [21], [24], [32], [33]. The within- and across-category tonal continua are equated in physical intervals, the decreased activation that was accompanied by a corresponding shift of activity to the mSTS/MTG areas concerned with phonological processing therefore results from the top-down influences of phonological representations through feedback mechanisms. The result indicates that there are dynamic interactions between the core auditory areas versus downstream regions involved with acoustic versus phonological processing in speech perception. This finding is consistent with the evidence from previous ERP and magnetoencephalography (MEG) studies which have shown that the onset of language specific phonological analysis occurs at 100∼200 ms. In this time window, the mismatch response (mismatch negativity and its MEG counterpart) is elicited by both acoustic and phonological variations, indicating that the processing of acoustic and phonological information is integrated at an early stage [34], [35]. Our results match up well with Hickok and Poeppel's dual-stream model of speech processing, according to which the projections from dorsal STG to mid-posterior STS/MTG are reciprocal and there are bidirectional connections between the left and right hemispheres at multiple levels [9]. Although the present study does not indicate that phonological properties can be extracted without some level of basic acoustic encoding, it does indeed suggest that there need not be a continuous relationship between low-level basic encoding and higher-level linguistic processing. When both acoustic and phonological features are present, it is possible that once a minimal amount of acoustic information is encoded, phonological processing dominants and modulates further detailed acoustic analysis in the core auditory areas through feedback mechanisms. This feedback mechanism is inconsistent with models that assume that phonological features are abstracted only after the high-level areas have received and analyzed all the required acoustic input from the low-level areas [7], [16].

Unlike previous research emphasizing task-specific top-down influences on the core auditory cortex [13]–[15], the present study reveals that processing of high-level information contained in the speech sound per se in the ventral brain regions can modulate activation elicited by low-level information in the dorsal brain regions. The result suggests that in speech perception there is a functional reorganization of auditory analysis induced by phonetic experience. That is, acoustic and phonological representations are computed in parallel, but phonological information is processed more efficiently and its activation exerts an inhibitory effect through feedback mechanisms on the concurrent auditory processing. The reorganization of auditory analysis is consistent with the existence of highly efficient network of phonological processing [36] and further indicates that there is immediate interaction between low-level acoustic and high-level phonological processing. The reorganization of auditory analysis is contingent on experience with the phonological features of a particular language, and once acquired, the language-specific phonological properties are automatically processed in speech perception, which modulates low-level acoustic processing. Speech and nonspeech sounds often contain multiple levels of information, for example, low-level spectral and temporal information and high-level experience-driven, context-dependent information. Further studies should explore nonspeech sounds to clarify whether such cortical dynamic interactions reflect a general mechanism for the processing of high-level and low-level information regardless of the linguistic status of the signal.

One issue that arises with the current study is whether phonological and acoustic processing took different pathways from the very beginning such that the across-category variation was treated entirely differently from the within-category variation. If that is actually the case, there should be no feedback mechanisms involved even if the interaction pattern is observed. The present data cannot rule out this possibility and further studies are needed to clarify the conflicting interpretations.

In conclusion, the present results are consistent with the functional hierarchy view of speech perception that assumes that the feedforward auditory pathway, the dorsal STG and lateral mSTS/MTG, are responsible for acoustic analysis and phonological processing, respectively. In addition, our results provide direct evidence on the cortical dynamics during the interaction of bottom-up acoustic analysis and top-down phonological representations, in which the acoustic analysis is modulated by phonological processing through feedback mechanisms. Our study demonstrates interactive dynamics underlying feedforward and feedback loops in speech perception, aligning well with Hickok and Poeppel's dual-stream model of speech processing [8], [9] that argues for reciprocal progression projections across brain regions.
